# Supplementary material for: The Antimicrobial Resistance Characteristics of Imipenem-Non-Susceptible, Imipenemase-6-Producing Escherichia coli
Source: Antibiotics (Basel). 2021 Dec 28;11(1):32. doi: 10.3390/antibiotics11010032 (PMC8772982; doi:10.3390/antibiotics11010032)
Supplement: Supplementary file 1 [file antibiotics-11-00032-s001.zip › antibiotics-1507660-supplementary 1.pdf]

Supplemental Table S1. Minimum inhibitory concentrations against antimicrobials among 42 isolates of *Escherichia coli*.

Strain No.	MIC (μg/mL)										
	IPM	MEPM	ETP	DRPM	PIPC	CAZ	CFPM	CPFX	LVFX	AMK	GM
ECL19-1	1 (S)	8 (R)	1 (I)	0.25 (S)	>64 (R)	>16 (R)	>16 (R)	>2 (R)	>4 (R)	<4 (S)	8 (I)
ECL19-2	2 (I)	32 (R)	16 (R)	2 (I)	>64 (R)	>16 (R)	>16 (R)	>2 (R)	>4 (R)	<4 (S)	4 (S)
ECL19-3	1 (S)	16 (R)	4 (R)	0.25 (S)	>64 (R)	>16 (R)	>16 (R)	>2 (R)	>4 (R)	<4 (S)	4 (S)
ECL19-4	2 (I)	16 (R)	2 (R)	1 (S)	>64 (R)	>16 (R)	>16 (R)	>2 (R)	>4 (R)	<4 (S)	8 (I)
ECL19-6	1 (S)	16 (R)	4 (R)	1 (S)	>64 (R)	>16 (R)	>16 (R)	>2 (R)	>4 (R)	<4 (S)	8 (I)
ECL19-7	1 (S)	16 (R)	4 (R)	0.25 (S)	>64 (R)	16 (R)	>16 (R)	>2 (R)	>4 (R)	16 (S)	2 (S)
ECL19-8	1 (S)	4 (R)	2 (R)	0.25 (S)	>64 (R)	>16 (R)	>16 (R)	>2 (R)	>4 (R)	<4 (S)	>8 (R)
ECL19-9	0.5 (S)	2 (I)	1 (I)	0.25 (S)	>64 (R)	>16 (R)	>16 (R)	>2 (R)	>4 (R)	<4 (S)	8 (I)
ECL19-10	0.5 (S)	4 (R)	4 (R)	2 (I)	>64 (R)	>16 (R)	>16 (R)	>2 (R)	>4 (R)	<4 (S)	8 (I)
ECL19-11	0.5 (S)	2 (I)	2 (R)	0.25 (S)	>64 (R)	>16 (R)	>16 (R)	>2 (R)	>4 (R)	<4 (S)	8 (I)
ECL19-12	1 (S)	8 (R)	2 (R)	0.5 (S)	>64 (R)	>16 (R)	>16 (R)	>2 (R)	>4 (R)	16 (S)	8 (I)
ECL19-13	1 (S)	16 (R)	8 (R)	1 (S)	>64 (R)	>16 (R)	>16 (R)	>2 (R)	>4 (R)	<4 (S)	>8 (R)
ECL19-15	1 (S)	8 (R)	1 (I)	0.25 (S)	>64 (R)	>16 (R)	>16 (R)	>2 (R)	>4 (R)	<4 (S)	4 (S)
ECL19-16	1 (S)	16 (R)	8 (R)	2 (I)	>64 (R)	>16 (R)	>16 (R)	>2 (R)	>4 (R)	<4 (S)	4 (S)
ECL19-17	1 (S)	16 (R)	16 (R)	16 (R)	>64 (R)	>16 (R)	>16 (R)	>2 (R)	>4 (R)	<4 (S)	8 (I)
ECL19-18	1 (S)	16 (R)	16 (R)	8 (R)	>64 (R)	16 (R)	>16 (R)	>2 (R)	>4 (R)	<4 (S)	8 (I)
ECL19-19	2 (I)	16 (R)	16 (R)	16 (R)	>64 (R)	>16 (R)	>16 (R)	>2 (R)	>4 (R)	<4 (S)	8 (I)
ECL19-20	4 (R)	32 (R)	32 (R)	32 (R)	>64 (R)	>16 (R)	8 (I)	>2 (R)	>4 (R)	<4 (S)	8 (I)
ECL19-22	2 (I)	16 (R)	32 (R)	16 (R)	>64 (R)	>16 (R)	>16 (R)	>2 (R)	>4 (R)	8 (S)	>8 (R)
ECL19-23	2 (I)	16 (R)	32 (R)	32 (R)	>64 (R)	>16 (R)	>16 (R)	>2 (R)	>4 (R)	8 (S)	8 (I)
M11	2 (I)	16 (R)	16 (R)	16 (R)	>64 (R)	>16 (R)	>16 (R)	>2 (R)	>4 (R)	<4 (S)	4 (S)
M20	2 (I)	8 (R)	16 (R)	8 (R)	>64 (R)	>16 (R)	>16 (R)	>2 (R)	>4 (R)	16 (S)	4 (S)

M24	1 (S)	16 (R)	16 (R)	8 (R)	>64 (R)	>16 (R)	>16 (R)	>2 (R)	>4 (R)	<4 (S)	8 (I)
M26	4 (R)	16 (R)	64 (R)	32 (R)	>64 (R)	>16 (R)	>16 (R)	>2 (R)	>4 (R)	16 (S)	8 (I)
M35	4 (R)	16 (R)	32 (R)	16 (R)	>64 (R)	>16 (R)	>16 (R)	>2 (R)	>4 (R)	8 (S)	8 (I)
M37	4 (R)	32 (R)	64 (R)	16 (R)	>64 (R)	>16 (R)	>16 (R)	>2 (R)	>4 (R)	<4 (S)	4 (S)
M39	1 (S)	8 (R)	16 (R)	2 (I)	>64 (R)	>16 (R)	>16 (R)	>2 (R)	>4 (R)	<4 (S)	8 (I)
M50	4 (R)	32 (R)	32 (R)	32 (R)	>64 (R)	16 (R)	>16 (R)	>2 (R)	>4 (R)	<4 (S)	>8 (R)
M54	4 (R)	32 (R)	64 (R)	32 (R)	>64 (R)	>16 (R)	>16 (R)	>2 (R)	>4 (R)	<4 (S)	>8 (R)
M55	2 (I)	16 (R)	32 (R)	8 (R)	>64 (R)	>16 (R)	>16 (R)	>2 (R)	>4 (R)	<4 (S)	>8 (R)
M56	2 (I)	16 (R)	32 (R)	8 (R)	>64 (R)	>16 (R)	>16 (R)	>2 (R)	>4 (R)	<4 (S)	<8
M58	2 (I)	16 (R)	32 (R)	16 (R)	>64 (R)	>16 (R)	>16 (R)	>2 (R)	>4 (R)	32 (I)	8 (I)
M60	2 (I)	32 (R)	64 (R)	32 (R)	>64 (R)	>16 (R)	>16 (R)	>2 (R)	>4 (R)	<4 (S)	>8 (R)
M64	2 (I)	16 (R)	32 (R)	16 (R)	>64 (R)	>16 (R)	>16 (R)	>2 (R)	>4 (R)	<4 (S)	>8 (R)
M65	2 (I)	32 (R)	64 (R)	32 (R)	>64 (R)	>16 (R)	>16 (R)	>2 (R)	>4 (R)	<4 (S)	>8 (R)
M66	1 (S)	1 (S)	8 (R)	4 (R)	>64 (R)	16 (R)	>16 (R)	<0.5 (S)	<0.5 (S)	<4 (S)	2 (S)
M69	1 (S)	8 (R)	16 (R)	8 (R)	>64 (R)	>16 (R)	>16 (R)	>2 (R)	>4 (R)	<4 (S)	8 (I)
M70	2 (I)	8 (R)	32 (R)	8 (R)	>64 (R)	>16 (R)	>16 (R)	>2 (R)	>4 (R)	<4 (S)	8 (I)
M73	1 (S)	4 (R)	32 (R)	8 (R)	>64 (R)	>16 (R)	>16 (R)	>2 (R)	>4 (R)	8 (S)	8 (I)
M75	4 (R)	32 (R)	64 (R)	32 (R)	>64 (R)	>16 (R)	>16 (R)	>2 (R)	>4 (R)	8 (S)	>8 (R)
M76	1 (S)	4 (R)	16 (R)	4 (R)	<8 (S)	8 (I)	<1 (S)	>2 (R)	>4 (R)	<4 (S)	4 (S)
M77	1 (S)	8 (R)	8 (R)	4 (R)	>64 (R)	>16 (R)	>16 (R)	>2 (R)	4 (R)	<4 (S)	4 (S)
