# Supplementary material for: The Antimicrobial Resistance Characteristics of Imipenem-Non-Susceptible, Imipenemase-6-Producing Escherichia coli
Source: Antibiotics (Basel). 2021 Dec 28;11(1):32. doi: 10.3390/antibiotics11010032 (PMC8772982; doi:10.3390/antibiotics11010032)
Supplement: Supplementary file 1 [file antibiotics-11-00032-s001.zip › antibiotics-1507660-supplementary 2.pdf]

Supplemental Table S2. The characteristics of carbapenemase and ESBL production among 42 isolates of *Escherichia coli*.

Strain No.	Carbapenemase phenotype	Carbapenemase gene	ESBL phenotype	ESBL genes	Plasmid replicon typing
ECL19-1	+	bla_{IMP-6}			FIA, FIB, F, I1, N
ECL19-2	+	bla_{IMP-6}			FIA, FIB, F, I1, N
ECL19-3	+	bla_{IMP-6}	+	$bla_{CTX-M-2}$, $bla_{CTX-M-14}$	FIA, FIB, F, I1
ECL19-4	+	bla_{IMP-6}			FIA, FIB, F, I1, N
ECL19-6	+	bla_{IMP-6}			FIA, FIB, F, I1, N
ECL19-7	+	bla_{IMP-6}			F, N
ECL19-8	+	bla_{IMP-6}			FIA, FIB, F, I1, N
ECL19-9	+	bla_{IMP-6}	+	-	FIA, FIB, F, I1, N
ECL19-10	+	bla_{IMP-6}	+	-	FIA, FIB, F, I1, N
ECL19-11	+	bla_{IMP-6}			FIA, FIB, F, I1, N
ECL19-12	+	bla_{IMP-6}	+	-	I1
ECL19-13	+	bla_{IMP-6}	+	-	FIA
ECL19-15	+	bla_{IMP-6}	+	-	FIA, FIB, F, I1, N
ECL19-16	+	bla_{IMP-6}	+	-	FIA, FIB, F, I1, N
ECL19-17	+	bla_{IMP-6}	+	-	FIA, FIB, F, I1, N
ECL19-18	+	bla_{IMP-6}	+	-	FIA, F, I1,
ECL19-19	+	bla_{IMP-6}	+	-	FIA, FIB, F, I1, N
ECL19-20	+	bla_{IMP-6}	+	-	FIA, FIB, F, I1, N
ECL19-22	+	bla_{IMP-6}			FIA, FIB, F, I1, N
ECL19-23	+	bla_{IMP-6}	+	-	FIA, FIB, F, I1
M11	+	bla_{IMP-6}	+	$bla_{CTX-M-2}$	FIA, FIB, N, F
M20	+	bla_{IMP-6}	+	$bla_{CTX-M-15}$	FIA, N, F, B/O

M24	+	*bla*$_{IMP-6}$	+	*bla*$_{CTX-M-2}$	FIA, N, F
M26	+	*bla*$_{IMP-6}$			FIA, N, F, B/O
M35	+	*bla*$_{IMP-6}$	+	*bla*$_{CTX-M-2}$, *bla*$_{CTX-M-15}$	FIA, N, F, B/O
M37	+	*bla*$_{IMP-6}$	+	*bla*$_{CTX-M-2}$	FIA, FIB, N, F
M39	+	*bla*$_{IMP-6}$	+	*bla*$_{CTX-M-2}$	FIA, FIB, N, F
M50	+	*bla*$_{IMP-6}$	+	*bla*$_{CTX-M-2}$	FIA, FIB, N, F
M54	+	*bla*$_{IMP-6}$	+	*bla*$_{CTX-M-2}$	FIA, N, F
M55	+	*bla*$_{IMP-6}$	+	*bla*$_{CTX-M-2}$	FIA, N, F
M56	+	*bla*$_{IMP-6}$	+	*bla*$_{CTX-M-2}$	FIA, N, F
M58	+	*bla*$_{IMP-6}$			FIA, FIB, N, F, A/C
M60	+	*bla*$_{IMP-6}$	+	*bla*$_{CTX-M-2}$	FIA, N, F
M64	+	*bla*$_{IMP-6}$	+	*bla*$_{CTX-M-2}$	FIA, N, F
M65	+	*bla*$_{IMP-6}$	+	*bla*$_{CTX-M-2}$	FIA, N, F
M66	+	*bla*$_{IMP-6}$	+	*bla*$_{CTX-M-2}$	FIB, N, F
M69	+	*bla*$_{IMP-6}$	+	*bla*$_{CTX-M-2}$	FIB, N, F
M70	+	*bla*$_{IMP-6}$			FIA, FIB, N, F
M73	+	*bla*$_{IMP-6}$	+	*bla*$_{CTX-M-2}$	FIA, FIB, N, F, B/O
M75	+	*bla*$_{IMP-6}$			FIA, FIB, N, F
M76	+	*bla*$_{IMP-6}$			FIB, F, A/C
M77	+	*bla*$_{IMP-6}$	+	*bla*$_{CTX-M-2}$	FIA, FIB, N, F
